# The efficacy of high-dose penicillin G for pneumococcal pneumonia diagnosed based on initial comprehensive assessment at admission: an observational study

**DOI:** 10.1186/s13104-018-3510-7

**Published:** 2018-06-20

**Authors:** Junpei Komagamine

**Affiliations:** Department of Internal Medicine, National Hospital Organization Tochigi Medical Center, 1-10-37, Nakatomatsuri, Utsunomiya, Tochigi 3208580 Japan

**Keywords:** Community-acquired pneumonia, High-dose penicillin, Pathogen-targeted therapy, *Streptococcus pneumoniae*

## Abstract

**Objectives:**

High-dose penicillin therapy is effective in approximately 90% of pneumococcal pneumonia cases diagnosed based on urinary pneumococcal antigen tests or Gram staining at admission. The efficacy of high-dose penicillin therapy for pneumococcal pneumonia diagnosed based on an initial comprehensive assessment comprising a syndromic approach, Gram staining of sputum and urinary pneumococcal antigen testing was investigated.

**Results:**

Seventy adult patients diagnosed with pneumococcal pneumonia based on an initial comprehensive assessment and treated with high-dose penicillin G at admission were included. The median patient age was 76.5 years, and 37.1% of the patients were women. The urinary pneumococcal antigen test was positive in 67.1% of all patients, and Gram staining of sputum showed that gram-positive cocci were dominant in 58.6% of the patients. The primary outcome was treatment success based on vital signs until day 6. Treatment with high-dose penicillin G was effective in 87.1% of the patients (95% CI 79.1–95.2%), and the proportion of patients who received other antibiotics because of treatment failure with penicillin G was only 5.7%. The efficacy of high-dose penicillin G treatment for pneumococcal pneumonia diagnosed based on a comprehensive assessment at admission may be comparable to that in previous reports.

**Electronic supplementary material:**

The online version of this article (10.1186/s13104-018-3510-7) contains supplementary material, which is available to authorized users.

## Introduction

In initial assessments of pneumonia cases, it is often difficult to identify the causative organism based on clinical presentation and microbial examination [1–8], and most pneumonia guidelines thus recommend initial empirical treatment using broad-spectrum antibiotics [9, 10]. However, previous randomized controlled trials have shown that pathogen-directed antibiotic therapy using narrow-spectrum antibiotics is as effective as empirical antibiotic therapy for community-acquired pneumonia (CAP) [11, 12]. Given that adverse drug events occur more frequently with empirical treatment than with pathogen-directed therapy [11, 13], the latter may be preferred.

*Streptococcus pneumoniae* is the most common causative organism of CAP requiring hospitalization in adults [14, 15]. Previous studies have reported high treatment success rates with high-dose penicillins or amoxicillin for pneumococcal pneumonia diagnosed based on either urinary pneumococcal antigen test [16, 17] or Gram staining of sputum [13] at admission. However, little is known about the efficacy of strategies to diagnose pneumococcal pneumonia based on an initial comprehensive assessment combining clinical history, Gram staining of sputum and the urinary pneumococcal antigen test followed by treatment with high-dose penicillins as an initial monotherapy. Thus, the aim of this research was to evaluate the efficacy of high-dose penicillin G for pneumococcal pneumonia diagnosed based on an initial comprehensive assessment at admission.

## Main text

### Methods

#### Study design and setting

This study was a retrospective observational study utilizing data obtained from electronic medical records of the National Hospital Organization Tochigi Medical Center, a 350-bed acute-care hospital in Japan. At that time, this hospital did not have an intensive care unit. This research was retrospectively registered as UMIN000025887 (January 28, 2017).

#### Participants and inclusion criteria

Patients aged 18 years or older who were admitted to the hospital for pneumonia and initially treated with high-dose penicillin G from April 2012 to May 2017 were included. During the study period, 78 patients who had received high-dose penicillin G treatment for pneumonia were identified. Among them, eight patients did not initially receive penicillin G treatment on the day of admission. Overall, 70 patients were included in the final analysis.

#### Initial comprehensive assessment and treatment

Beginning in April 2012, the hospital started pathogen-targeted therapy for hospitalized adult pneumonia patients in routine practice. When pneumonia is diagnosed by medical history, a physical examination, and a chest X-ray, expectorated sputum samples are collected by a nurse before administering antibiotics. If possible, physicians or laboratory technicians then perform and interpret sputum Gram staining at admission. Only good-quality sputum is used to determine the causative pathogen, although sputum quality is rather subjective and at the discretion of the principal physicians. When *S. pneumoniae* is clinically suspected to be the causative organism of pneumonia at the initial assessment, physicians perform urine antigen testing for *S. pneumoniae*. The physicians also prescribe either high-dose penicillin G or ampicillin at their discretion if one of the following is present: (1) gram-positive diplococci upon Gram staining; (2) positive urine antigen test; (3) sudden onset of fever, chest pain, lobar infiltrate, and leukocytosis at presentation (syndromic approach). Our syndromic approach is similar to a strategy used by a pathogen-directed therapy group in a previous study [11]. Patients who were treated with ampicillin based on the initial comprehensive assessment at admission were excluded because ampicillin is often prescribed empirically for aspiration pneumonia in this hospital.

### Microbiological investigations

In routine practice, physicians perform sputum Gram staining and establish a sputum culture when possible at admission; during the diagnostic process, physicians also establish at least two sets of blood cultures. When *S. pneumonia* is clinically suspected to be the causative organism of pneumonia, physicians perform urine antigen tests using the BinaxNOW *S. pneumoniae* urinary antigen kit (Alere Scarborough, Inc. Scarborough, USA). The proportion of patients who were evaluated using these tests is shown in Additional file 1. The definitions of sputum quality for Gram staining, predominant morphotype, and etiology of pneumonia are also shown in Additional file 2.

### Data collection and measurement

Information on age, gender, symptoms, swallowing problems, past medical history, results of laboratory testing, and chest radiographs was retrieved from electronic medical records at admission. The pneumonia severity index (PSI) [18] and CURB-65 score [9, 19] at admission were calculated.

The primary outcome was the treatment success rate. Based on a previous study [12], treatment was judged to be successful when clinical stability was achieved between 2 and 6 days after admission (detailed information is provided in Additional file 2). Secondary outcomes included in-hospital mortality, 30-day mortality, 30-day readmission rate, and adverse drug events caused by penicillin G. Adverse drug events were determined based on documented physician diagnosis in medical records. The rate of change in the use of other intravenous antibiotics due to a lack of effectiveness against pneumonia was also assessed, as the clinical stability of pneumonia patients based on vital signs can be delayed, even if treatment is effective [20]. The last follow-up date was May 31, 2017.

#### Statistical analysis

Originally, this study was planned to target 100 patients, a size similar to pathogen-targeted therapy groups in previous randomized controlled trials [11, 12]. However, penicillin G use for pneumococcal pneumonia at this hospital has declined since 2015, partly because ampicillin was more frequently selected as an initial antibiotic agent for pneumococcal pneumonia due to the inconvenience of prescribing penicillin G. Therefore, the outcomes of 70 patients were analyzed.

Baseline characteristics were examined using descriptive statistics. Primary and secondary outcomes were calculated as the proportion of patients in whom those outcomes occurred in all patients. The Chi squared test was used to compare primary outcomes between subgroups of patients for whom pneumonia severity was classified based on PSI or CURB-65. The Chi squared test was also used for comparison of primary outcomes between pneumococcal pneumonia and pneumonia of undetermined etiology based on microbial investigation. Given that *S. pneumoniae* is the leading cause of pneumonia of unknown etiology [21, 22], a similar efficacy of treatment between each subgroup was hypothesized. These analyses were performed using Excel statistical software package version 2.11 (Bellcurve for Excel; Social Survey Research Information Co., Ltd., Tokyo, Japan); the level of significance was set at 5%.

### Results

The baseline characteristics of the patients are presented in Table 1. The median age of the 70 patients was 76.5 years, and 26 (37.1%) were women. The median PSI score of the pneumonia cases was 97.5 (Table 2).

**Table 1:** Baseline characteristics of 70 patients with pneumococcal pneumonia diagnosed based on an initial comprehensive assessment at admission

Characteristics	Total^a^
	N = 70
Patient characteristics	
Age, median (IQR)	76.5 (68.0–85.8)
Women	26 (37.1)
Health-care associated	11 (15.7)
Institutional resident	11 (15.7)
Pneumococcal vaccination	9 (12.9)
Number of medications, median (IQR)	4 (1–7)
Immunosuppressive drug use^b^	5 (7.1)
Dysphagia^c^	18 (25.7)
Prehospital antibiotic use	23 (32.9)
Past medical history	
Heart failure	3 (4.3)
Ischemic heart disease^d^	3 (4.3)
Stroke or TIA	14 (20.0)
Diabetes mellitus	13 (18.6)
Chronic pulmonary disease^e^	18 (25.7)
Liver disease	2 (2.9)
Dementia	18 (25.7)
Chronic kidney disease	6 (8.6)
Active cancer	0 (0.0)
Current smoker, n (%)	7 (10.0)
Regular drinker, n (%)	20 (28.6)
Patient symptoms, n (%)	
Acute onset (< 4 days)	43 (61.4)
Chest pain	18 (25.7)
Fever	57 (81.4)
Chill	14 (20.0)
Hemoptysis	2 (2.9)
Cough	49 (70.0)
Sputum	40 (57.1)
Throat pain	7 (10.0)
Vital signs, median (IQR)	
Temperature, °C	38.4 (37.4–39.0)
Systolic blood pressure, mmHg	130.0 (110.0–146.5)
Diastolic blood pressure, mmHg	70.5 (63.0–84.3)
Heart rate per minutes	103.5 (90.0–117.0)
Respiratory rate per minutes^f^	24 (20–28)
Oxygen saturation at room air, %	91 (88–94)

^a^Values are shown as numbers (percentages) unless stated otherwise

^b^This value includes corticosteroids, chemotherapy and immunosuppressive drugs

^c^Dysphagia was judged based on an assessment by speech therapists during a hospital stay

^d^Ischemic heart disease included angina, myocardial infarction and coronary artery graft surgery

^e^Chronic pulmonary disease included asthma, chronic obstructive pulmonary disease, and bronchiectasis

^f^Among 63 patients

**Table 2:** Characteristics and management of 70 pneumococcal pneumonia cases diagnosed based on an initial comprehensive assessment at admission

Characteristics	Total^a^
	N = 70
Radiological characteristics	
Bilateral infiltrate	15 (21.4)
Upper lobe infiltrate	10 (14.3)
Pleural effusion	2 (2.8)
Microbial characteristics	
Gram stain	
GPC dominant (good-quality only)^b^	24 (34.3)
GPC dominant (regardless of quality)	41 (58.6)
Not performed	11 (15.7)
Urine pneumococcal antigen	47 (67.1)
Blood culture	
*Streptococcus pneumoniae*	5 (7.1)
*Streptococcus mitis*	1 (1.4)
*Staphylococcus aureus*	1 (1.4)
Presumptive etiology	
Undetermined	17 (24.3)
*Streptococcus pneumoniae*	46 (65.7)
Other pathogens	7 (10.0)^c^
Pneumonia severity	
Pneumonia severity index	
Total score, median (IQR)	97.5 (77.3–121.0)
Class 1	5 (7.1)
Class 2	7 (10.0)
Class 3	19 (27.1)
Class 4	26 (37.1)
Class 5	13 (18.6)
CURB-65	
Low risk	22 (31.4)
Intermediate risk	23 (32.9)
High risk	25 (35.7)
Management during hospital stay	
Penicillin G treatment	
Continuous infusion	65 (92.9)
Duration, day, median (IQR)	7.0 (4.3–7.0)
Switch to oral amoxicillin	10 (14.3)
Macrolide combination	0 (0.0)
Corticosteroid use	2 (2.9)
Tracheal intubation	1 (1.4)
Vasopressor use	1 (1.4)

^a^Values are shown as numbers (percentages) unless stated otherwise

^b^Sputum specimens were judged to be high quality if there were fewer than 10 squamous epithelial cells and greater than 10 polymorphonuclear cells per low-power field

^c^This value includes *Haemophilus influenzae* (four cases), *Staphylococcus aureus* (one case), *Streptococcus mitis* (one case), and *Streptococcus agalactiae* (one case)

In the initial assessment, the urinary pneumococcal antigen test was positive in 47 (67.1%) of the 70 patients, and gram-positive cocci (GPC) were dominant upon Gram staining of sputum samples from 41 patients (58.6%). Regarding the etiology of pneumonia based on the final results of microbial investigation, 46 cases (65.7%) were *Streptococcus pneumoniae*, and 17 (24.3%) were of an undetermined etiology. Among the 46 cases of presumed pneumococcal pneumonia, penicillin-susceptible *S. pneumoniae* (PSSP) and penicillin-intermediate resistant *S. pneumoniae* (PISP) were isolated from 20 patients and one patient, respectively. Penicillin-resistant *S. pneumoniae* (PRSP) was not isolated from any patient.

The treatment success rate was 87.1% (Table 3). The proportion of patients who were switched from penicillin G to other antibiotics due to pneumonia treatment failure (detailed information is described in an Additional file 2) was only 5.7%. Two patients died in the hospital. During the study period, no drug adverse events caused by penicillin G were documented. However, in one patient, penicillin G was replaced by high-dose ampicillin because continuous infusion of penicillin G was judged by the principal physician to be difficult due to delirium. The treatment success rate did not differ significantly among the subgroups of patients classified according to pneumonia severity and was not different between pneumococcal pneumonia and pneumonia of undetermined etiology based on the final results of microbial investigations (detailed information is described in an Additional file 1).

**Table 3:** Clinical outcomes of 70 pneumococcal pneumonia cases diagnosed based on clinical judgment at admission

Characteristics	Number of patients (% [95% CI])
	Total, N = 70
Primary outcome	
Clinical success until day 6^a^	61 (87.1 [79.1 to 95.2])
Secondary outcomes	
Adverse drug events due to penicillin G	0 (0.0 [0.0 to 0.0])
Switch to other antibiotics^b^	4 (5.7 [0.1 to 11.3])^b^
30-day mortality	1 (1.4 [− 1.4 to 4.3])^c^
In-hospital death	2 (2.9 [− 1.1 to 6.9])^c, d^
Readmission within 30 days	1 (1.4 [− 1.4 to 4.3])

^a^Clinical success was defined as the condition in which all the following threshold values were achieved for a 24-h period: temperature, ≤ 37.2 °C; heart rate, ≤ 100 beats/min; respiratory rate, ≤ 24 breaths/min; systolic blood pressure, ≥ 90 mmHg; oxygen saturation, ≥ 90% or arterial oxygen partial pressure, and ≥ 60 mmHg when the patient was not receiving supplemental oxygen

^b^This value includes cases in which penicillin G was replaced by other antibiotics because of pneumonia treatment failure (more detailed information was described in an Additional file 2)

^c^This patient died due to hospital-acquired pneumonia after recovery from community- acquired pneumonia

^d^This patient died due to the complication of acute respiratory distress syndrome in influenza A pneumonia derived from an in-hospital influenza outbreak

### Discussion

The results of this study showed that treatment with high-dose penicillin G for pneumococcal pneumonia diagnosed based on a comprehensive initial assessment using a syndromic approach, Gram staining of sputum, and the urinary pneumococcal antigen test was effective in 87% of all cases. The results of the present study are similar to those of past studies, showing that pathogen-directed therapy [11–13, 16, 17] or empirical therapy with broad-spectrum antibiotic agents [11, 12, 20–25] is effective in 80–90% of CAP patients, even though the definition of treatment success differed somewhat from those of past studies [26]. Furthermore, adverse drug events in the present study were less frequent than in past studies that investigated the efficacy of broad-spectrum antibiotic agents for CAP patients [11, 12, 22–25]. Given that sputum specimens of high quality are not often obtained [14] and that the sensitivity of the urinary pneumococcal antigen test for diagnosis of pneumococcal pneumonia is inadequate [4], these findings are notable. Furthermore, this strategy may be preferable because drug adverse events occur in empirical therapy more frequently than in pathogen-targeted therapy [11, 13].

Nonetheless, these findings should be interpreted with caution given that this study lacked a control group, which is a major limitation. Although the baseline patient characteristics and the severity of pneumonias in the present study were not different from those in past studies of CAP [11–13, 16, 17, 20–25], the lack of a control group may confound the outcomes. Furthermore, the 95% confidence interval for the primary outcome was wide due to the small sample size, and its lower limit was less than 80%. This efficacy rate might be unacceptable. Therefore, a well-designed study is needed to evaluate the efficacy of this strategy for pneumonia patients.

The efficacy of high-dose penicillin G therapy did not differ between pneumococcal pneumonia and pneumonia of undetermined etiology based on the final results of the microbial investigations. Given that penicillin G is a narrow-spectrum antibiotic agent, this finding supports past studies showing that *S. pneumoniae* is the leading cause of pneumonia of unknown etiology [27, 28], although some cases of pneumonia of undetermined etiology in this study might have been caused by viral infection [29].

De-escalation therapy after initial empirical therapy is uncommon in practice due to the undetermined etiology in most pneumonia patients [14]. Furthermore, relative to the practice of treating pneumonia patients with empirical antibiotic therapy, residents should be educated in the practice of estimating the suspected causative pathogen using clinical history and microbial tests [30, 31]. Therefore, further studies are warranted to identify an effective strategy for estimating the causative organism of pneumonia at an initial assessment and to treat the causative organism with narrow-spectrum antibiotic agents.

### Conclusions

The efficacy of high-dose penicillin G therapy for pneumococcal pneumonia diagnosed based on a comprehensive assessment at admission may be acceptable. Nonetheless, the results of this study should be interpreted with caution due to the lack of a control group. A well-designed study is needed to evaluate the efficacy of this strategy for pneumonia patients.

## Limitations

First, this study had a retrospective observational design. Second, this study was limited to a single center and to hospitalized patients receiving high-dose penicillin G therapy at admission; thus, the results may not be easily generalizable. These findings should be confirmed by future studies performed at other institutions. Third, it was unclear how often patients who were initially diagnosed with pneumococcal pneumonia at admission were treated with other antibiotic agents, such as ampicillin and ceftriaxone. It was also unclear whether the physicians prescribed penicillin G for pneumonia patients at admission because they truly suspected pneumococcal pneumonia. Fourth, PRSP was rarely isolated in this hospital. Therefore, these results may not be generalizable to other hospitals in which PRSP is more prevalent. Fifth, it is inconvenient to administer penicillin G via intravenous infusion, and high-dose ampicillin might be preferable as an initial antibiotic agent for pneumococcal pneumonia. Sixth, the study hospital does not have an intensive care unit. Therefore, these findings might not be applicable to cases of very severe pneumococcal pneumonia. Seventh, data were collected from usual care. Given that adverse drug events are often unrecognized by physicians [32], these outcomes might be underestimated. Finally, a statistical analysis to investigate predictive factors associated with treatment success was not conducted.

## Additional files

**Additional file 1: Table S1.** Enforcement and documentation rates of vital signs, microbial testing and imaging at admission. ^a^Sputum specimens were judged to be high quality if there were fewer than 10 squamous epithelial cells and greater than 10 polymorphonuclear cells per low-power field. **Table S2.** Clinical outcomes according to the severity of pneumonia based on CURB-65 and the pneumonia severity index. ^a^Clinical success was defined as the condition in which all the following threshold values were achieved for a 24-h period: temperature, ≤ 37.2 °C; heart rate ≤ 100 beats/min; respiratory rate ≤ 24 breaths/min; systolic blood pressure, ≥ 90 mmHg; and oxygen saturation ≥ 90% or arterial oxygen partial pressure ≥ 60 mmHg when the patient was not receiving supplemental oxygen. ^b^Comparison of outcomes between subgroups according to severity of pneumonia was performed using the Chi squared test. **Table S3.** Comparison of primary outcomes between subgroups according to the presumptive etiology of pneumonia based on the final results of microbial investigation. ^a^Clinical success was defined as the condition in which all the following threshold values were achieved for a 24-h period: temperature ≤ 37.2 °C; heart rate ≤ 100 beats/min; respiratory rate ≤ 24 breaths/min; systolic blood pressure, ≥ 90 mmHg; and oxygen saturation ≥ 90% or arterial oxygen partial pressure ≥ 60 mmHg when the patient was not receiving supplemental oxygen. ^b^Comparison of outcomes between subgroups was performed using the Chi squared test.

**Additional file 2: Text S1.** Definition of terms and outcomes used in this study. **Text S2.** Four cases in which penicillin G was replaced by other antibiotics because of treatment failure for pneumonia.

## Abbreviations

CAP: community-acquired pneumonia
CI: confidence interval
GPC: gram-positive cocci
HCAP: healthcare-associated pneumonia
IQR: interquartile range
PRSP: penicillin-resistant *S. pneumoniae*
PISP: penicillin-intermediate resistant *S. pneumoniae*
PSI: pneumonia severity index
PSSP: penicillin-susceptible *S. pneumoniae*
TIA: transient ischemic attack
