# Supplementary material for: The efficacy of high-dose penicillin G for pneumococcal pneumonia diagnosed based on initial comprehensive assessment at admission: an observational study
Source: BMC Res Notes. 2018 Jun 20;11:399. doi: 10.1186/s13104-018-3510-7 (PMC6011604; doi:10.1186/s13104-018-3510-7)
Supplement: Supplementary file 1 — Additional file 1: Table S1. Enforcement and documentation rates of vital signs, microbial testing and imaging at admission. aSputum specimens were judged to be high quality if there were fewer than 10 squamous epithelial cells and greater than 10 polymorphonuclear cells per low-power field. Table S2. Clinical outcomes according to the severity of pneumonia based on CURB-65 and the pneumonia severity index. aClinical success was defined as the condition in which all the following threshold values were achieved for a 24-h period: temperature, ≤ 37.2 °C; heart rate ≤ 100 beats/min; respiratory rate ≤ 24 breaths/min; systolic blood pressure, ≥ 90 mmHg; and oxygen saturation ≥ 90% or arterial oxygen partial pressure ≥ 60 mmHg when the patient was not receiving supplemental oxygen. bComparison of outcomes between subgroups according to severity of pneumonia was performed using the Chi squared test. Table S3. Comparison of primary outcomes between subgroups according to the presumptive etiology of pneumonia based on the final results of microbial investigation. aClinical success was defined as the condition in which all the following threshold values were achieved for a 24-h period: temperature ≤ 37.2 °C; heart rate ≤ 100 beats/min; respiratory rate ≤ 24 breaths/min; systolic blood pressure, ≥ 90 mmHg; and oxygen saturation ≥ 90% or arterial oxygen partial pressure ≥ 60 mmHg when the patient was not receiving supplemental oxygen. bComparison of outcomes between subgroups was performed using the Chi squared test. [file 13104_2018_3510_MOESM1_ESM.pdf]

Table S1. Enforcement and documentation rates of vital signs, microbial testing and imaging at admission.

	**Total, N = 70**
Vital sign, n (%)	
Consciousness	70 (100.0)
Temperature	70 (100.0)
Respiratory rate	63 (90.0)
Systolic blood pressure	70 (100.0)
Diastolic blood pressure	70 (100.0)
Heart rate	70 (100.0)
Oxygen saturation	70 (100.0)
Laboratory tests, n (%)	
White-cell count	70 (100.0)
Hematocrit	70 (100.0)
Hemoglobin	70 (100.0)
Sodium	70 (100.0)
Potassium	70 (100.0)
Urea nitrogen	70 (100.0)
Creatinine	70 (100.0)
Glucose	62 (88.6)
Blood gas	26 (37.1)
Microbial tests, n (%)	
Sputum culture	
Total	62 (88.6)
Only good quality[a]	30 (42.9)
No documentation about quality	6 (8.6)
Sputum Gram staining	
Total	59 (84.3)
Only good sputum quality[a]	30 (42.8)
No documentation about quality	3 (4.3)
No documentation about detailed information regarding morphotype	21 (30.0)
Blood culture	63 (90.0)
Urine pneumococcal antigen	67 (95.7)
Imaging test, n (%)	
Chest X-ray	70 (100.0)

Table S2. Clinical outcomes according to the severity of pneumonia based on CURB-65 and the pneumonia severity index.

**Severity of pneumonia**	**Clinical success until day 6[a], (%)**	***P*-value[b]**
CURB-65		0.63
Low risk, N = 22	18/22 (81.8)	
Intermediate risk, N = 23	21/23 (91.3)	
High risk, N = 25	22/25 (88.0)	
Pneumonia Severity Index		0.60
Class 1, N = 5	5/5 (100.0)	
Class 2, N = 7	5/7 (71.4)	
Class 3, N = 19	16/19 (84.2)	
Class 4, N = 26	23/26 (88.5)	
Class 5, N = 13	12/13 (92.3)	

Table S3. Comparison of primary outcomes between subgroups according to the presumptive etiology of pneumonia based on the final results of microbial investigation.

**Outcome**	**Undetermined N = 17**	***Streptococcus pneumoniae* N = 46**	***P*-value[b]**
Clinical success until day 6[a], n (%)	15 (88.2)	40 (87.0)	0.89
