# Supplementary material for: The efficacy of high-dose penicillin G for pneumococcal pneumonia diagnosed based on initial comprehensive assessment at admission: an observational study
Source: BMC Res Notes. 2018 Jun 20;11:399. doi: 10.1186/s13104-018-3510-7 (PMC6011604; doi:10.1186/s13104-018-3510-7)
Supplement: Supplementary file 2 — Additional file 2: Text S1. Definition of terms and outcomes used in this study. Text S2. Four cases in which penicillin G was replaced by other antibiotics because of treatment failure for pneumonia. [file 13104_2018_3510_MOESM2_ESM.pdf]

Text S1. Definition of terms and outcomes used in this study.

Pneumonia:
A diagnosis of pneumonia was defined based on the presence of new infiltrates on a chest X-ray and one or more of the following symptoms: presence of fever (> 38.0ºC), hypothermia (< 36.0ºC), dyspnea, cough (with or without sputum), and chest pain.
High-dose penicillin G:
Administration of high-dose penicillin G was defined as 12 million or more units of penicillin G per day, adjusted as needed based on the patient's renal function.
Health-care-associated pneumonia:
Cases of health-care-associated pneumonia (HCAP) included patients who had any of the following characteristics: (1) hospitalized in an acute-care hospital for two or more days within the past 90 days; (2) resided in a nursing home or long-term care facility; (3) received intravenous antibiotic therapy for other reasons, including chemotherapy or wound care, within the past 30 days; or (4) attended a hospital or a hemodialysis clinic (*Am J Respir Crit Care Med* 2005;171:388-416).
Quality of sputum specimens:
For Gram staining of sputum, specimens were judged to be good quality if there were fewer than 10 squamous epithelial cells and greater than 10 polymorphonuclear cells per low-power field, independent of the presence of a predominant morphotype based on a previous study (*BMC Infect Dis* 2014;14:534).
Predominant morphotype:
A predominant morphotype was considered to be present when Gram staining revealed bacteria mostly or exclusively corresponding to one Gram morphotype. For missing data regarding detailed information on a morphotype of more than gram-positive coccus (GPC), "GPC unspecified" was used.
Presumptive etiology of pneumonia:
There is no universally accepted gold standard for determining the definite etiology of pneumonia. Based on recent studies (*Thorax* 2005;60:672-8, *BMC Infect Dis* 2014;14:534), the etiology of pneumonia was considered presumptive in this study if one of the following criteria was met: (1) blood cultures yielded a bacterial pathogen in the apparent absence of an extrapulmonary focus; (2) cultures of pleural fluid or transthoracic needle aspiration fluids yielded a bacterial pathogen; (3) moderate or heavy growth from semi-quantitative cultures of high-quality sputum yielded a bacterial pathogen in the apparent absence of other causative pathogens; (4) a positive urine antigen test for *S. pneumoniae* in the apparent absence of other causative pathogens. *S.*

pneumoniae organisms isolated from sputum and blood samples were classified into the three categories: penicillin-susceptible *S. pneumoniae* (PSSP; MIC ≤ 2.0 μg/dl), penicillin-intermediate resistant *S. pneumoniae* (PISP; MIC ≈ 4.0 μg/dl), and penicillin-resistant *S. pneumoniae* (PRSP; MIC ≥ 8.0 μg/dl) (*MMWR Morb Mortal Wkly Rep* 2008;57(50):1353-5). The etiology of pneumonia was classified according to three main etiological categories: *S. pneumoniae*, other pathogens, or undetermined.

Dysphagia:

Patients were determined to have a swallowing problem if they fulfilled the following: (1) known dysphagia prior to admission; or (2) dysphagia based on assessment by swallowing experts during a hospital stay. At this hospital, swallowing problems are routinely screened and assessed by experts for all hospitalized patients, regardless of dysphagia risk.

Clinical stability:

Clinical stability was defined as a condition in which all the following threshold values were achieved for a 24-hour period: temperature of ≤ 37.2°C, heart rate of ≤ 100 beats/min, respiratory rate of ≤ 24 breaths/min, systolic blood pressure of ≥ 90 mmHg, and oxygen saturation of ≥ 90% or arterial oxygen partial pressure of ≥ 60 mmHg when the patient was not receiving supplemental oxygen (*Thorax* 2010;65:101-6). For most pneumonia patients in usual care, nurses at this hospital documented vital signs three or more times a day, and the outcome was evaluated retrospectively based on these vital signs.

Text S2. Four cases in which penicillin G was replaced by other antibiotics because of treatment failure for pneumonia.

Patient 1:
An elderly man. After admission, his respiratory status was unchanged or slightly improved by penicillin G treatment. However, his respiratory status suddenly worsened after the aspiration of foods on the fourth hospital day. Therefore, the principal physician stopped his meals and changed penicillin G to ampicillin-sulbactam.

Patient 2:
An elderly man. After admission, his respiratory status worsened despite penicillin G treatment. A diagnosis of acute exacerbation by chronic obstructive pulmonary disease occurred on the second hospital day. Corticosteroid, inhaled bronchodilator, and meropenem were started.

Patient 3:
An elderly man. After admission, his respiratory status was unchanged despite penicillin G treatment. However, his respiratory status suddenly worsened due to pneumothorax on the second hospital day. Therefore, a chest tube was inserted, and ceftriaxone and azithromycin were started.

Patient 4:
A middle-aged woman. After admission, her respiratory status was unchanged despite penicillin G treatment. Her respiratory status worsened slightly due to an unknown cause on the third hospital day. Therefore, penicillin G was replaced by ceftriaxone and azithromycin.
